# The PRO-CAR-T™ study: achieving excellence in patient and public involvement and engagement

**DOI:** 10.1186/s41687-026-01036-x

**Published:** 2026-03-24

**Authors:** Karen L. Shaw, Sarah E. Hughes, Olalekan L. Aiyegbusi, John Ansell, Evelyn Chakera, Ronjon Chakraverty, Mark Chatterley, Paul Ferguson, Foram Khatsuria, Rebecca Lloyd, Christel McMullan, J. Devin Peipert, Lester Pyatt, Vladyslava Yarosh, Melanie J. Calvert

**Affiliations:** 1 Centre for Patient Reported Outcome Research (CPROR), Department of Applied Health Sciences, Murray Learning Centre, University of Birmingham, Birmingham, B15 2TT UK; 2 National Institute of Health and Care Research (NIHR) Blood and Transplant Research Unit in Precision Cellular Therapeutics, Universities of Birmingham and Oxford, Birmingham and Oxford, UK; 3 NIHR Applied Research Collaboration West Midlands, Birmingham, UK; 4 Birmingham Health Partners Centre for Regulatory Science and Innovation, University of Birmingham, Birmingham, UK; 5 NIHR Birmingham Biomedical Research Centre, Birmingham, UK; 6 MRC Molecular Haematology Unit, Radcliffe Department of Medicine, MRC Weatherall Institute of Molecular Medicine, University of Oxford, Oxford, UK; 7 NIHR Oxford Biomedical Research Centre, Oxford, UK; 8 University Hospitals Birmingham NHS Foundation Trust, Birmingham, UK

**Keywords:** Patient reported outcomes, Patient reported outcome measures, Patient and public involvement, Public engagement, Advanced therapies, Advanced therapy medicinal products, Haematology, Quality of life assessment, Health-related quality of life

## Abstract

**Background:**

Patient and Public Involvement and Engagement (PPIE) is advocated in Patient Reported Outcome (PRO) research, but implementation remains inconsistent and often suboptimal. Many barriers exist, including a lack of understanding about how to apply the principles of PPIE in practice. This paper offers an example of how PPIE can be meaningfully integrated into the development of a digital PRO system within the complex and rapidly evolving field of advanced therapies. The aims are to offer practical insights about how PPIE was conceptualised and operationalised in the context of this study, and use the lessons learned to inform future PPIE strategies for PRO research.

**Methodology:**

Co-produced strategy and delivery plans for PPIE were developed by stakeholders, including patients, carers and members of the public, and drawing on national standards and best practice guidelines. This was followed by activities to recruit additional Patient and Public Partners, provide training, identify opportunities for PPIE and map these to key decision points in the research plan. The success of these strategies and activities was evaluated using a range of reflective and assessment methods, in which personal experiences and formal feedback were combined to assess impact and identify areas for improvement.

**Results:**

Evaluation revealed that PPIE was successfully integrated across project management, study conduct, and dissemination, generating both research value and personal benefits. However, several conceptual, methodological, and practical challenges were encountered. Three key themes emerged: (i) the challenges of distinguishing between research and PPIE in PRO studies (ii) the factors that shape meaningful PPIE and research impact and (iii) areas for improvement. Early planning, finding the right people, and working co-productively were identified as critical facilitators.

**Conclusions:**

Integrating excellent PPIE is achievable in PRO research, even in challenging contexts that involve life-threatening conditions and complex emerging therapies. Although barriers exist, our experience suggests many can be addressed and several actionable recommendations and resources are offered to strengthen PPIE in future PRO research.

## Background

Patient and Public Involvement and Engagement (PPIE) is a term that has specific meaning for organisations like the National Institute for Health and Care Research (NIHR). *Involvement* is defined as “research ‘with’ or ‘by’ people who use services rather than ‘to’, ‘about’ or ‘for’ them”, and *engagement* as the varied ways that “information and knowledge about research is provided and disseminated” [1]. These definitions reflect the ‘intrinsic values’ that underpin all health research and care (e.g. trust, transparency and accountability) and ‘instrumental values’ that ensure research is relevant, feasible and inclusive [2].

PPIE is clearly relevant to Patient Reported Outcome (PRO) research, given its focus on understanding health outcomes directly from the patient’s perspective. This includes studies that seek to conceptualise PROs, develop robust measurement tools, establish systems to collect PRO data in research and clinical settings, or address challenges related to implementation. The importance of PPIE is widely advocated by regulatory authorities [3], researchers [4–6], developers [7], and patients themselves [8]. However, much of this discourse focuses on the inclusion of patients and the public in the development of patient reported outcome measures (PROMs), especially the stages of concept elicitation, item generation, or psychometric testing; with interviews and focus groups commonly recommended to ascertain stakeholder views. Less attention has been paid to how patients and the public can work *alongside* researchers to shape the design and conduct of this research, or the value of PPIE in PRO research more broadly [9–12].

The literature also highlights numerous barriers that hinder meaningful PPIE in PRO research. These challenges include lack of clear guidance, insufficient training, limited understanding of patient and public roles, resource constraints and uncertainties about suitable methods [8–10, 13, 14]. Others have also observed that PPIE is often conflated or confused with *patient participation* in PRO research (i.e., where individuals take part in a research study as a ‘subject’) [10, 13, 14]. Although these distinctions may seem ‘semantic’, they have important implications [14]. For example, UK regulatory frameworks set out different expectations and processes for research and PPIE, particularly around safeguarding. Notably, research involving human participants requires formal ethical review, whereas PPIE generally does not. This is because patients and the public are considered to be collaborative partners with the research team, rather than participants, and therefore do not need to be protected from research risks [15]. This does not imply that PPIE is without risk; the ethical implications should always be considered, especially when working with vulnerable groups. However, managing the boundaries between research and PPIE is known to be challenging [13, 14].

Unfortunately, these many barriers have led to inconsistent and suboptimal practices, resulting in the underutilisation of patient and public contributions in PRO research. While there are indications of improvement, a 2017 scoping review found that patient and the public views are integrated into PRO research only in limited ways. In fact, a quarter of studies reported no evidence of patient or public input of any kind – be it participation, involvement or engagement [5].

Addressing these challenges has been central to the PRO-CAR-T™ study. This study sits within NIHR Blood and Transplant Research Unit in Precision Cellular Therapeutics (BTRU-PCT); a research programme that seeks to improve therapies and treatment for blood cancer and blood disorders [16]. The specific aim of the PRO-CAR-T™ study is to develop a new digital platform to capture PROs for patients receiving chimeric antigen receptor T-cell (CAR-T) therapy [17]. CAR-T is considered a ‘breakthrough’ Advanced Therapy Medicinal Product (ATMP) for patients with blood cancer and represents a rapidly growing focus for clinical practice and research, as outlined in Box 1.

**Table:** Box 1 Overview of ATMPs and CAR-T therapy

**Advanced Therapy Medicinal Therapies (ATMPs)** are groundbreaking therapies that use cells, genes, or tissues to treat the root cause of disease [18]. While most have focused on rare conditions that have no effective treatment, or where treatment can fail, ATMPs are increasingly targeting long-term conditions, injury and prevention [18]. As such, they offer new hopes for diverse patients, including those with cancer, neurodegenerative, inherited, autoimmune, metabolic, musculoskeletal, immune, ocular and hearing disorders; with more medicinal products and therapeutic targets in the pipeline [18]. Many can be given as a single treatment, and some are potentially curative. As such, ATMPs have the potential to transform the lives of patients and revolutionise care models. It is unsurprising, therefore, that the ATMP sector is showing rapid growth in the number of developers, clinical trials, regulatory approvals and patients being treated [19–25] .
**Chimeric antigen receptor T-cell (CAR-T) therapy** is a ‘breakthrough’ ATMP that has made significant strides in development and regulation [26]. It has been approved for patients with specific blood cancers, including some lymphomas, leukaemias, and multiple myeloma [27, 28] and is typically given to patients with refractory or relapsed disease (i.e. after other treatments have failed). CAR-T involves genetically modifying a patient’s T-cells in a laboratory to express chimeric antigen receptors (CARs) on their surface. These receptors are designed to recognise and bind to specific proteins on cancer cells. The modified T-cells are multiplied and, when sufficiently abundant, infused back into the patient to seek out and destroy cancer cells [27]. This targeted and personalised approach to treatment is showing promising remission rates [28–30]. However, while CAR-T is offering hope to many patients, it is also associated with high patient burden, including treatment-related toxicities that can be life-threatening and require early intervention [31, 32]. There are also uncertainties about the long-term impacts of CAR-T and unwarranted variations in patient outcomes that need better understanding [33]. Not all patients respond to treatment [30] and there are uncertainties about whether CAR-T therapy can cause new malignancies [34]. Despite these concerns, use of CAR-T therapy continues to grow, reaching a cumulative total of 9039 patients treated across Europe in 2022; representing a 10-fold increase since 2018 [35]. Research is also expanding, aimed at improving efficacy, reducing side effects, and widening its application to other cancers and autoimmune diseases.

The need to fully understand the impact of ATMPs is highlighted by the UK Stem Cell Strategic Forum, which explicitly advocates the use of PROMs, stating that “collecting, and publishing, patients’ self-reported quality of life outcomes data, in a consistent and thorough way, is urgently needed” [33]. Indeed, while ATMPs are providing new therapeutic possibilities, their novelty and complexity mean they may also have safety risks that are known, unknown or unforeseen. Regulation is having to advance rapidly to meet these challenges and emphasises the importance of assessing safety and efficacy throughout the ATMP Pathway [36]. This includes well-designed clinical trials and post-licence monitoring (e.g. 15 years) [37]. However, despite recommendations to include PROMs in ATMP trials [3, 33, 38], they are known to be underused [39–41]. This underutilisation may stem from several factors: lack of knowledge about the most suitable outcomes for these new generation therapies [39], absence of validated measures [39], uncertainties about the appropriateness of existing instruments [41], scepticism about their clinical value [42] or concerns about patient burden [43]. There are calls, therefore, for improved stakeholder engagement, including PPIE, to ensure PROMs for patients receiving ATMPs are relevant and fit for purpose.

This article focuses on the implementation of PPIE for the PRO-CAR-T™ study. The aims are to offer practical insights about how PPIE was conceptualised and operationalised in the context of this study, and use the lessons learned to inform future PPIE strategies for PRO research. It provides details on how PPIE was integrated throughout the different research stages and synthesises the perspectives of patients, carers, the public, PPIE specialists, clinicians, and researchers to offer practical strategies for others involved in PRO research; especially those working in the complex and rapidly evolving field of advanced therapies.

## Methods

The project involved several stages where patients and the public worked alongside researchers to (i) design the research, (ii) establish the foundations for PPIE, (iii) develop and implement PPIE for the PRO-CAR-T™ study and (iv) learn from experience and outcomes.

### Designing the research

The PRO-CAR-T™ study [17] is part of the National Institute of Health and Care Research (NIHR) Blood and Transplant Research Unit in Precision Cellular Therapeutics (BTRU-PCT) [16]. The BTRU-PCT includes multiple studies that range from basic science to applied health research, underpinned by a common goal to improve patient experiences of precision-cellular therapies and outcomes. Patients and the Public were invited to shape the pre-funding bid for the BTRU-PCT via an online meeting; supported by existing PPIE Leads in the applicants’ organisations who helped recruit individuals with lived experience of blood cancer and/or advanced therapies already known to them. The aim was to include people with a range of different perspectives, but to also ensure discussions could happen in a timely manner, knowing that their involvement support needs had already been assessed and met. This included six patients; two family members, including a parent of a child with a rare disease; and one plasma/blood donor. The meeting was facilitated by a co-applicant with expertise in PPIE. The intention was to ensure patient and public perspectives informed the research programme and PPIE plan. Those involved were invited to remain involved, if successfully funded.

As one of the proposed projects, the PRO-CAR-T™ study aimed to develop a new digital platform for capturing PROs to remotely monitor symptoms, side effects, and health-related quality of life in patients with blood cancer undergoing CAR T-cell therapies; specifically for use in routine clinical practice, but with potential for broader application in ATMP trials. Thus, an important component is to assess feasibility for use in the UK’s National Health Service (NHS). Following best practice [44–47], the study was planned with a mixed-methods multi-phase design, and included work to develop an electronic PRO system (Work Package 1); build and test system usability (Work Package 2); and evaluate feasibility of the digital system when deployed in a clinical setting (Work Package 3).

### Establishing the foundations for PPIE

Following funding approval, a series of deliberate actions were untaken to establish a robust and enabling foundation for PPIE; designed to underpin all studies within the BTRU-PCT research programme, including the PRO-CAR-T™ study. These efforts were driven by best practice frameworks in PPIE [1, 48] that recommend principles and implementation strategies for high quality PPIE.

Key activities included early involvement of Patient and Public Partners; allocation of dedicated resources, including specialist staff to manage PPIE; fostering shared understanding of PPIE principles amongst professional and lay members, including clarification of their roles and responsibilities; ensuring inclusive opportunities for PPIE; ongoing support and training; and implementing evaluation mechanisms to facilitate continuous learning (see Table 1). This strategy was underpinned by a model of co-production; characterised by mutual respect for different expertise, power sharing, collaborative decision-making and the joint creation of new knowledge [49].

**Table 1:** Foundations of the PPIE Strategy for the BTRU-PCT research programme

Key principles	Aims and key activities	Main outcomes
Early involvement	Online meeting held with patients and public members to develop initial research bid, including PPIE plans. Recruitment facilitated by existing PPIE networks and session led by co-applicant experienced in PPIE. Contributors invited to remain involved if successfully funded.	Ensured funded research bid focused on issues important to patients and the public, and had a PPIE plan that was integrated throughout the research cycle, responsive to the needs of potential Patient and Public Partners, and adequately resourced. Also ensured a ready formed group of interested patients and public contributors to support the research once funded, enabling their involvement in the study set-up.
PPIE management	PPIE Manager employed 3-days per week, recognising PPIE is a skilled and time-consuming endeavour [52], is not well established in ATMP research [53], and some BTRU-PCT researchers may have limited PPIE experience.	PPIE Manager served as a central resource, ensuring PPIE was appropriately planned, delivered, and assessed. Enabled training opportunities for staff/students, to enhance their PPIE competencies.
Shared purpose	Online meeting and home-based activities undertaken to co-produce a Strategy document [50] and Delivery Plan [51] to clarify ambitions for PPIE and actions needed to realise these. Recognised that the BTRU-PCT is a cross-disciplinary and multi-agency organisation, with geographically dispersed staff who have varied PPIE experience.	Helped align everyone’s understanding of PPIE and provided an evaluative framework on which to measure progress. Also helped ensure PPIE initiatives were underpinned by best practices [1], value-based frameworks [49] and national standards [48].
Co-production	PPIE based on a model of co-production [49], emphasising respect for different sets of expertise, shared decision-making and power-sharing. Patient and Public Partners were represented at all levels of BTRU-PCT management. ‘Working together’ was a standing agenda item to identify opportunities for shared decision-making, knowledge production, output development, dissemination and engagement. To promote a partnership ethos, joint workspaces (e.g. online file-sharing, digital noticeboards), information channels (e.g., newsletter) and opportunities for whole-group working (e.g., annual meetings where everyone can attend and contribute) were created.	Patient and Public Partners are not just consulted as individuals with lived experiences but seen as trusted partners who support mutual learning and benefit. The personal skills, expertise and knowledge of Patients and Public Partners are frequently offered, utilised and celebrated. They are represented in major events, meetings and outputs (e.g., presenting at annual meetings, attending meetings with funders, co-authoring reports and papers, co-designing and attending engagement events). Their contributions are formally acknowledged in ways that suit Patient and Public Partners (e.g., authorship, named acknowledgments, photos) to showcase personal involvement and promote wider public understanding of the research and PPIE.
Inclusive opportunities	Recruitment of Patient and Public Partners facilitated through contact with relevant hospital departments, charities, websites promoting involvement opportunities and online patient networks; following best practice guidance to support inclusion and diversity [1]. Varied involvement options offered to suit different preferences, including a PPIE Strategy Group (meet regularly with researchers and plan PPIE), PPIE Mailing Group (receive newsletter with opportunities for involvement that can usually be done from home with minimal preparation) and engagement activities/mechanisms to share work publicly and act as a gateway to involvement. Activities involve varied formats, venues and times, and where possible, offer multiple ways to be involved in an activity. Payments for time and out-of-pocket expenses are offered, to ensure cost is not a barrier to involvement; based on NIHR guidance [54].	Research is informed by diverse Patient and Public Partners, with varying backgrounds and experiences. Groups include a range of ages, ethnicities, socioeconomic statuses, occupations (student, working, retired, and caring roles), health levels (active treatment, in remission, long-term management) and protected characteristics (e.g., disability, sexuality). A flexible approach to involvement has enabled researchers to work in depth with Patient and Public Partners or consult widely, using various formats to support inclusion such as online meetings, in-person meetings, interactive workshops, home-based tasks, and simple polls. It has also enabled patients and the public to inform the research through different levels of commitment, from single contacts at one-off engagement events to regular involvement; offering both continuity and diversity.
Training, support and development	Training, support and development opportunities offered on an ongoing basis – in response to needs assessments and requests. Early activities were designed to welcome and orientate Patient and Public Partners, identify personal requirements, discuss roles and support available. Included signposting to useful information (e.g. Public Information Pack [55] and bespoke guides to support local working arrangements (e.g., payments, online meetings). Handbooks were also designed for staff [56] and those involved in PhD research; acknowledging that excellent resources for PPIE exist, but can be challenging to find and apply when experience is limited. Patient and Public Partners have opportunities to develop their roles and suggest new priorities for working together.	Early training and support acknowledged the different starting points of staff and Patient and Public Partners, helping develop shared understandings about roles, responsibilities, best practice, and the skills required to implement this knowledge effectively. Our co-productive approach means that learning is a two-way process, with staff offering opportunities to learn about their methods, and Patient and Public Partners helping to plan and deliver researcher training (e.g., using plain language). Our resources are shared publicly to facilitate wider learning PPIE. Many researchers and Patient and Public Partners have taken up opportunities for personal development (e.g., attending PPIE training, applying for patient scholarships to attend international conferences).
Impact	The experience and impact of PPIE in the PRO-CAR-T™ study are subject to continuous evaluation. Progress and impact of PPIE are benchmarked against out Delivery Plan and in relation to other formal assessment frameworks (e.g., PIRIT [57]). A range of formal and informal feedback mechanisms are used to review PPIE, allowing different opportunities to reflect on experiences and impact. E.g. Patient and Public Partners and researchers are invited to provide anonymous feedback after meetings, PPIE is a standing item on Project Management Group meetings, Researchers and Patient/Public Partners work together to prioritise outcomes and how to disseminate them (such as what to highlight in annual meetings). Have a formal complaints procedure, including independent contact.	Our impact log demonstrates a growing network of diverse Patient and Public Partners who individually and collectively add value to the BTRU-PCT’s governance, PPIE strategy and research. There has also been a demonstrable shift in the balance of power with Patients and Public Partners expressing an increasing sense of ownership and some have moved into leadership roles (e.g., co-chairing meetings, joining the management group). This is resulting in research impacts that extend beyond knowledge sharing, to include tangible outcomes such as patient-led videos, co-authored articles, co-created engagement tools.

Collectively, these initiatives created outcomes that created the conditions for meaningful partnership working, and the co-production of an agreed Strategy for the BTRU-PCT programme [50] and Delivery Plan [51] that described the practical steps required to achieve our ambitions (Table 1).

### Developing and implementing PPIE for the PRO-CAR-T^TM^ study

Having developed a general foundation for PPIE, it was possible to develop more tailored PPIE plans that aligned with the aims of individual projects, including a project-specific plan for the PRO-CAR-T™ study. This process began with an online ‘welcome’ meeting to introduce the study to Patient and Public Partners, followed by a ‘planning’ meeting to explore the proposed methods in more depth, identify opportunities for PPIE and map them to critical stages in the study. The researchers also held a hybrid training event aimed at enhancing understanding of PRO research, including key issues such as the equitable development and application of PROMs, and opportunities to ask questions. The aim was to develop a set of agreed activities where PPIE could add value to the research and create impacts driven by patient benefit. During this period, ongoing efforts were made to recruit additional Patient and Public Partners, with particular emphasis on increasing representation of individuals with lived experience of CAR T-cell therapy and enhancing the overall diversity of the group. Recruitment was facilitated through engagement with local hospital departments, national charities, patient involvement websites, online patient networks and community events, following best practice guidance on promoting inclusion and diversity [1].

### Evaluation: Learning from experiences and outcomes

Evaluation is a core component of our PPIE strategy, and comprises multiple feedback and assessment mechanisms designed to critically examine experiences, values, and interactions, and to foster continuous learning and improvement, including:

- Structured meeting reflections, captured through monthly study team meetings, quarterly director-level project management group meetings with patient representatives, bi-monthly online sessions with Patient and Public Partners, and annual funder meetings.
- Anonymous feedback surveys administered following key PPIE and research events.
- Informal feedback channels, such as email correspondence, supported by an open invitation for dialogue around working practices and improvement.
- PPIE activity logs, used to monitor patterns of engagement.
- Evaluation frameworks, including a modified version of the Public Involvement Impact Assessment Framework (PIRIT) [57] used to assess alignment with the UK Standards for Public Involvement in Research and our internal Delivery Plan [48].

In addition, contributors to the PRO-CAR-T™ study were invited to reflect on their experiences of PPIE, with an explicit purpose of sharing our collective insights to support PRO researchers through a co-authored publication. Reflections were guided by a set of open-ended questions exploring what individuals valued about our approach, what supported their involvement, challenges encountered, suggestions for future practice, and advice for researchers new to PPIE.

Responses were summarised into descriptive themes by the PPIE Manager (KLS), in discussion with the Lead Researcher (SH) and synthesised with findings from the existing assessments and feedback. From this integrated analysis, key issues of interest and concern were identified, including recommendations for best practice. All contributors informed the draft structure and content of this manuscript, which was reviewed and co-authored to reflect shared insights. It includes direct quotations from Patient and Public Partners to preserve the authenticity of lived experience and ensure their voices remain central. Other quotes have also been included to emphasise issues, as seen through varied lenses.

### Ethics

Research ethics is not required for PPIE, as Public Contributors are deemed collaborators rather than ‘research participants’ [15]. Nevertheless, permission is routinely sought for using identifiable data, such as names, photographs and quotes in publicly accessible materials.

A favourable research ethics committee approval was given for the research components of the PRO-CAR-T™ study (Health and Social Care Research Ethics Committee B (REC B), Reference: 23/NI/0104). The application detailed the role of PPIE for information only – as approval for PPIE was not required.

## Results

### Contributors

Diverse team members were involved and co-authored the paper, including Patient and Public Partners (*n* = 4), Professors who specialise in PRO research (*n* = 2), Research Fellows (*n* = 3), Professor of Haematology and Programme Director (*n* = 1), Programme Manager (*n* = 1), PPIE Academic Lead (*n* = 1), PPIE Manager (*n* = 1), and clinical collaborators (*n* = 2), who identified several topics relevant to high quality PPIE in PRO Research.

### PPIE activities

**Fig. 1:**
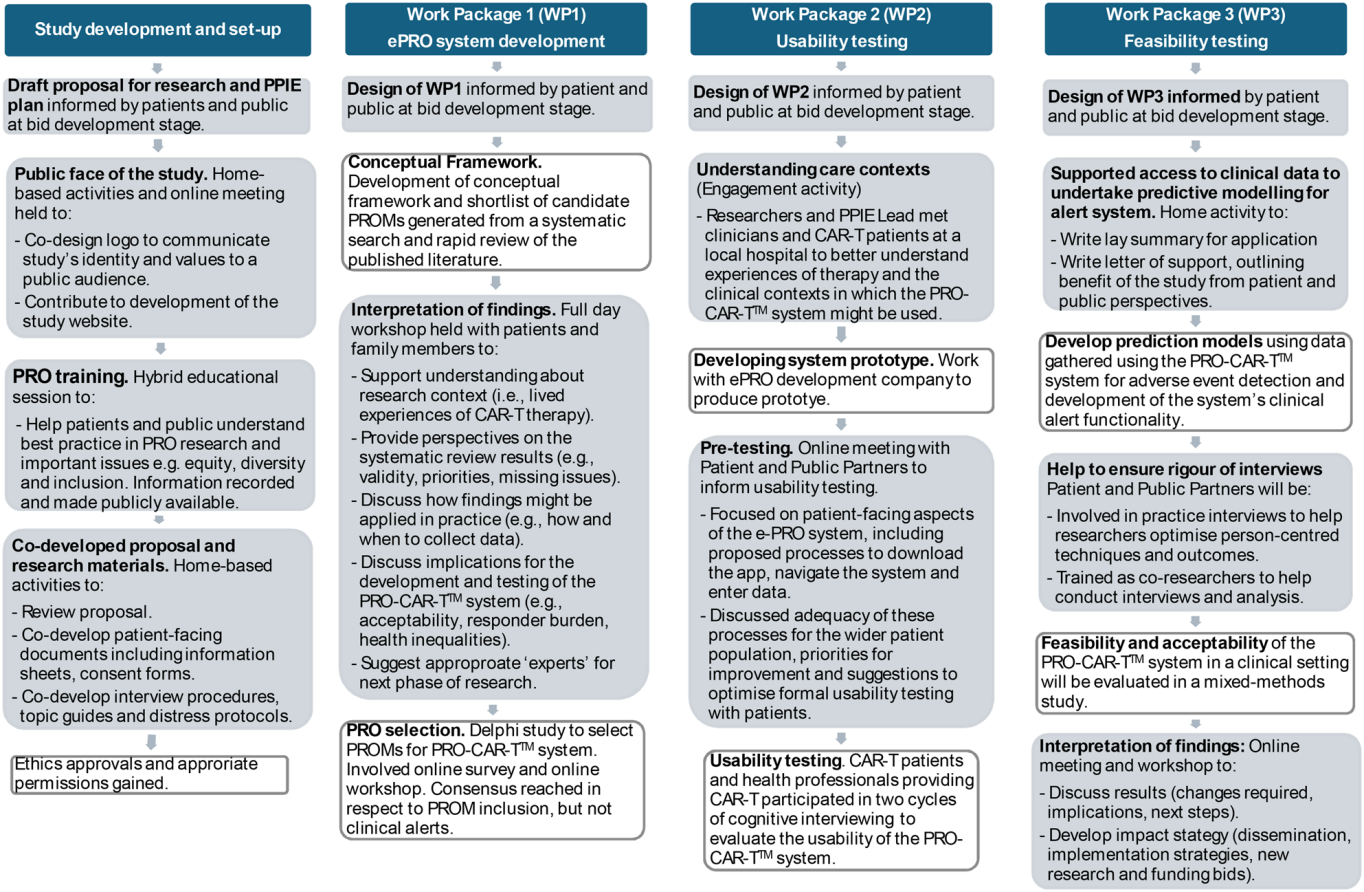
PPIE activities mapped to PRO-CAR-T™ study Work Packages. Key: Grey boxes=PPIE activity; White boxes= research activity. WPs 1 and 2 completed, WP 3 currently active

Our approach to conceptualising and operationalising PPIE for the PRO-CAR-T™ study resulted in a series of research-focused PPIE activities designed to ensure patients and the public shared decision-making at critical junctures and informed the study conduct (summarised in Fig. 1). Activities employed a variety of approaches tailored to the specific tasks and to offer diverse ways for people to be involved, whether collectively in meetings and workshops, or independently at their own time and pace.

These activities began with refining the pre-grant proposal into a detailed protocol suitable for ethical review and co-creating public-facing materials, including recognisable study logos to convey an appropriate public tone, interview schedules, distress protocols and patient information and consent forms (study set-up). This was followed by in-person workshops, online meetings and ‘at home tasks’ designed to support the development of the ePRO system (Work Package 1). This involved creating opportunities to deepen understanding of the lived experience of blood cancer and CAR-T, gain patient and public perspectives on the results of literature reviews and contextualise them in relation to people’s lives, and inform usability testing (Work Package 2). More recently, PPIE activities have guided feasibility testing in clinical settings, with plans to involve Patient and Public Partners as co-analysts to better understand patient data and optimise their use to further develop the PRO-CAR-T™ system (Work Package 3).

Several other PPIE activities have been integrated throughout the study. Patient and Public Partners have contributed to the steering and governance of the PRO-CAR-T™ study, as members of the BTRU-PCT Project Management Group which convenes quarterly to review progress, address challenges and ensure impact. They have also contributed to ongoing engagement and dissemination activities, including several co-authored journal articles and lay disseminations (e.g. newsletter features, videos and social media channels). Patient and Public Partners have also played a vital role in community engagement, including the design of informational and art-based posters, flyers and interactive activities aimed at raising awareness about the study and facilitating two-way conversations about PRO research in ATMPs. Work is also underway to co-produce ‘a route to impact’ strategy, informed by the PRO impact metrics identified by Rivera et al., 2019 [58] and guided by the Evidence-based Model for the exchange and Transfer of Research Knowledge (EMTReK) to enhance stakeholder involvement [59].

### Patient and Public Partners

These efforts have been shaped by a growing number of Patient and Public Partners who have become involved through our various engagement strategies. Although PPIE membership has fluctuated slightly, the group has steadily grown from the orininal pre-grant group of contributors (*n* = 9), to reach a maximum of 25 individuals, to date. This group includes patients with lived experience of various blood cancers and/or advanced therapies, family members and donors. Demographically, the group is broad, encompassing men and women across a wide age range (18–25 to 74–85 year groups), from diverse ethnic and socioeconomic backgrounds. The life circumstances of Patient and Public Partners are equally varied, including students, full- and part-time workers, homemakers, retirees and carers. Several participants also have protected characteristics. While involvement is not geographically restricted, most Patient and Public Partners live in the two UK cities where the research is based or surrounding areas. That said, the regions have differing geographical and demographic profiles.

Levels of involvement across PPIE activities has varied; as determined by the requirements of the individual activities and the preferences and competing demands of Patient and Public Partners. Meeting attendance is typically 8 and 12 members, including some who attend consistently, and others who contribute on a more ad hoc basis.

### Reflections and lesson learnt

Although the study remains ongoing, many components have been successfully completed and reported through papers co-authored with Patient and Public Partners [60, 61]. The added value of PPIE to the research produced has also been widely recognised. For instance, experts in oncology, have commented on the work, noting that “the integration of concepts beyond symptom management in the framework is novel, and probably a testament to the inclusion of patient and caregiver perspectives, and should be commended” [62].

Our own evaluation of the strategies employed, progress achieved, and challenges encountered has highlighted several key themes related to (i) the challenges of distinguishing between research and PPIE in PRO research (ii) the factors that shape meaningful PPIE and research impact and (iii) areas for improvement.

#### The challenges of distinguishing between research and PPIE

Our findings highlighted the importance of work undertaken to define PPIE in the context of this project and how this was central to developing an integrated PPIE strategy. Indeed, whilst developing our PPIE strategy, we acknowledged the ongoing debates about the diverse understandings related to PPIE, including the evolving language and definitions used to reflect shifts in how these roles and relationships are conceptualized [63]. Thus, an ongoing challenge was delineating PPIE activities from those more appropriately considered research and requiring ethical approval. Reflecting back, several steps proved helpful. Firstly, we adopted the NIHR definitions of ‘involvement,’ ‘engagement,’ and ‘participation,’ ensuring that Patient and Public Partners also understood these distinctions [1]. We also examined relevant literature and guidance to identify recommendations for involving patients and the public in PRO research. Using self-assessment tools and relevant taxonomies and checklists, we determined whether proposed activities were best categorised as research or PPIE [15, 63–65]. We also reviewed frameworks developed to support PPIE in PRO research, but found only one specific to the development of PRO measures [9]. Based on this, we categorised our activities as falling into one of the following categories, shown in Table 2. However, the distinctions between research and PPIE were of less concern to Patients and Public Partners, whose primary focus was to support the research.

**Table 2:** Our approach to managing the boundaries

Activity and purposes	Ethical requirements (UK)	Examples
**Engagement** Share research with patients and the public to support two-way learning, support societal debate, build trust in research, invite people to get involved in research, inspire young people to become the next generation of researchers.	*Ethical/regulatory approval*: Not usually required, but ethical issues should be considered, and activities should use ethical practices* *Our case*: Did not seek ethical review for engagement activities but requested formal consent to use identifiable data (e.g., photos, quotes, demographic information) in public facing materials such as websites, newsletters and articles – and followed relevant data protection guidelines. Adhered to organiser’s arrangements when attending external engagement events.	Engagement can use many approaches to target different audiences: • Share information about the PRO research through social media, newsletters, traditional media such as radio/television. • Attend research fairs, open-days, cultural events. • Share information in the community with specific groups, in public spaces or through creative arts. • Work with relevant local and national charities to share information via their patient and public facing networks. • Find opportunities to share information through relevant health organisations, and care teams, including patient experience teams where these exist. *For example*: We shared information about the study and opportunities for involvement with a range of charities and hospitals using flyers co-created with patients and public partners. We also attended family community events, using co-produced art and interactive activities to help explain our research.
**Involvement** Provide patient and public perspectives on research to inform its commissioning, design, conduct, interpretation and dissemination. Seek to include a range of perspectives with emphasis on diversity.	*Ethical/regulatory approval*: Not usually required, but ethical issues should be considered, and activities should use ethical practices. May be some exceptions, e.g. public contributors are in direct contact with study participants or their data, such as conducting research interviews or analysing transcripts.* *Our case*: Did not seek ethical review for involvement activities, but did request formal consent to use identifiable data (e.g., photos, quotes, demographic information) in public-facing materials such as websites, newsletters and articles. Plans for PPIE were also described in ethical/regulatory applications for transparency.	Involvement can occur throughout the research life-cycle including: • Priority setting: Involved with research commissioners, funders or regulatory boards, industry or priority setting initiatives to identify and prioritise PRO research. • Management: Involved in study steering groups to support decision-making. • Research design: Inform plans to develop, validate or use PROMs, and examine other PRO-related research questions by sharing perspectives on feasibility of study designs; contexts in which PROs likely to be used e.g., cultural issues, impact of condition on using PROMs, health inequalities, potential benefits to patients. • Research conduct: Inform, review, or co-produce research materials (e.g., patient information, interview topic guides) and ethical applications, facilitate recruitment, act as peer researchers, support interpretation of findings. • Dissemination: Inform or co-design knowledge transfer plans, co-author research articles, co-present findings, co-develop lay/public-facing messaging. • Help develop or review new research bids. *For example*: Our Patient and Public Partners provided lived experience insights into the CAR-T patient/carer journey to inform development of research materials (e.g., interview/workshop topic guides) and to provide early steering around key design requirements to maximise uptake and acceptability of the digital PRO collection for patients.
**(Stakeholder) Research** Patients and public work with research team in a collaborative manner to answer specific research questions using research methods. Seeks views from a defined sample. Generates findings that may have wider application.	*Ethical/regulatory approval*: Usually required. Likely to be 'involvement' when asking about issues that inform research design, conduct and interpretation – including how best to ensure research is relevant and feasible to participants and potential end users. In contract, likely to be considered 'research' if you wish to record and share the public contributor’s responses to the questions, or if you require specific information about an individual. *Our case*: Did not seek ethical approval when patient and public contributors worked with researchers to inform their decision-making (e.g., understanding the lived experiences of patients to better understand the relevance of PROs or discussing factors that may impact on patients abilities to report PROs or use patient apps). However, deemed research when individual responses were recorded and reported as evidence-based answers to specific research questions (e.g., What level of consensus is there for including a specific PRO, do patients interpret the questions as intended by the researcher?).	Stakeholder research typically uses qualitative research methods (e.g., focus groups and cognitive interviewing) and consensus-building techniques (e.g., Delphi method, nominal group method) to answer specific research questions that have been informed by PRO development frameworks and/or seek to generate theory. May use surveys. *For example*: Sense-checking findings from a literature review of symptoms and wider impacts of CAR T-cell therapies was undertaken as a PPIE activity; whereas a formal Delphi consensus study to select PROMs for the PRO-CAR-T system was classed as a research participation opportunity.
**(Testing) Research** Provide data to answer specific research question. Likely to include strict eligible criteria, specific sampling frameworks.	*Ethical/regulatory approval*: Usually required. Patients and members of the public are involved as ‘subjects’ to provide answers to specific research questions. *Our case*: Ethical approval was sought to recruit patients and carers for usability testing and the feasibility study. Patient and Public Partners informed these studies, but did not participate in them.	Testing research typically includes: psychometric testing to evaluate PROM measurement properties, implementation studies (e.g., pilot, feasibility and effectiveness studies), concept elicitation, cognitive debriefing, and content validation studies as part of PRO measure development. *For example*: Patient and Public Partners helped develop the research designs, including advising on patient recruitment strategies and co-creating patient information and consent forms. They will also support interpretation of findings. However, they do not participate in the studies.

N.B. This is only suggestive, and decisions will depend on local and national governance arrangements and processes. Always check with funders and employing research organisations

*May require criminal checks if it involves unsupervised activities with vulnerable groups. In our case: all researchers and PPIE lead had Disclosure and Barring Service (DBS) clearance as a requirement of undertaking their research

To aid clarity, we also followed recommendations from the literature to use distinct labels to distinguish PPIE activities from the research components of the PRO-CAR-T™ study (e.g. using PPIE *approaches* instead of research *methods*, *group* *discussions* instead of *focus groups*, *contributions* instead of *data*) [13, 14].

Given our Patient and Public Partners were considered part of the team, there was no expectation that they would particate in the PRO-CAR-T™ study. That said, we were mindful of the small patient population in CAR-T and we did not want to act as barriers or gatekeepers to participation where patient or carers demonstrated an interest in taking part in the research elements of the study (e.g. Delphi study). However, invitations were only extended after evaluating the potential for bias and ensuring that their participation was unlikely to confound the results.

#### Factors that shaped meaningful PPIE and research impact

Several factors seemed particularly important in shaping meaningful impact that added value to the research and created impactful outcomes. These included (i) early planning and integration (ii) finding the right people and supporting them appropriately, and (iii) co-productive working.

##### Early planning and integration

Collaborating with Patient and Public Partners from the outset allowed time to understand the lived experiences of patients and their families, and integrate these insights into the PRO-CAR-T™ study. Early involvement and training, with ongoing opportunities for questions, also provided time to help Patient and Public Partners understand their roles within the study and learn about the basic premises and methods in PRO research; enhancing the quality of interactions. This helped foster a sense of ownership and investment among Patient and Public Partners.

*Baking PPIE in from the beginning works wonders. If the whole project is built and developed with the idea of PPIE then those views and involvement can seamlessly flow into the project. Bolting on PPIE to an existing project*,* or just having it as an afterthought*,* can lead to people not feeling they are actually helping*,* creating disillusionment and eventually ending up with no interaction or poor interaction. [Patient Partner]*

Additionally, we discovered that activities perceived by researchers as brief and straightforward, such as voting for a preferred logo and suggesting design modifications, played important roles in relationship-building. These simple tasks served as an accessible entry point for patients and members of the public, especially those previously unfamiliar with PPIE. Engaging in joint decision-making allowed them to make meaningful contributions to the research process. Acting on their suggestions early also demonstrated key co-production principles, including shared-decision-making and mutual respect for different knowledge forms; which in turn, fostered a foundation of trust between patient and public contributors, and researchers.

*My advice to researchers is start with something small to test the waters. Maybe have someone look over a document. Or just set up a meeting with a patient to learn about their life. I think once a patient has started to be involved it is clear to see how they can help*,* even if that help is just to humanise the research that is happening. [Patient Partner]*

##### Finding the right people and supporting them appropriately

Patient and Public Partners were recruited through various mechanisms, emphasising inclusion and diversity. Our pre-grant group (n = 9) had a reasonable level of diversity (e.g. patients, carers, donors, parents; demographic characteristics) and all agreed to remain involved after funding. However, we recognised that certain groups, such as individuals from ethnic minorities and patients with very recent experience of CAR-T, were underrepresented. To address this, we collaborated with charities (e.g., South Asian Health Foundation) and local hospitals to broaden diversity, and engaged with under-represented groups at community events. This approach not only ensured that ‘real-world’ issues (e.g., respondent burden, health inequalities) remained at the forefront, but also helped better understand the context in which the ePRO system would be implemented. For example, involving people with experiences of standard cancer treatment, those in the first wave of CAR-T, and those with very recent treatment experiences allowed the team to consider their findings in the context of the rapidly changing healthcare landscape and its implications for the PRO-CAR-T™ system. However, including diverse individuals requires a flexible and supportive approach, recognising that people have competing demands, medical needs, and different preferences for involvement.

*It is clear that consideration for patients is at the heart of how a lot of the involvement is set up. For instance*,* having breaks in the meetings*,* which seems like such a small thing*,* but really can make the difference between being able to be involved or not [Patient Partner].*

Accordingly, we offered various types of activities and ways of working and acknowledged that while many people will contribute regularly, others will have variable or limited involvement (by choice or necessity). To address this, we developed a regular newsletter to recognise people’s contributions, keep them updated about progress, and offer open invitations to remain involved. This was supplemented by additional contact from the PPIE Manager and Study Leads to ensure more personalised engagement, recognising that building and maintaining relationships is critical to sustaining PPIE in the long term.

*My thoughts and comments are properly considered and actively sought out. I don’t feel like I have been involved just to tick a box. [Patient Partner]*

It became clear early on that careful attention to personal needs was also crucial. This realisation emerged during a meeting where lunch had been planned a ‘short distance’ from the meeting room, but proved difficult for those with limited mobility. Since then, we have implemented more comprehensive accessibility assessments to eliminate physical obstacles, enhance communication, and promote inclusivity. For instance, we ensure the use of accessible meeting spaces, run accessibility checks on documents, consider dietary, medical, and cultural requirements, offer overnight accommodation when travel is likely to act as a barrier, and organize family-friendly meetings to support parents and carers. However, while our approach has worked for most people, with some increasing their level of involvement, it did not work for everyone, with some patient partners perhaps feeling less connected and involved than others. Understanding how to better support individuals (and having the resources to do so) is an ongoing challenge.

##### Co-productive working

The key to effective PPIE and co-production [49] lies in fostering strong relationships built on trust, respect, and cooperation. Experiential learning has proven particularly effective in breaking down barriers between researchers and Patient and Public Partners by providing opportunities to visualise PPIE, develop skills, and observe real-time results. Relationship-building activities, such as annual meetings and workshops where researchers, patients, and family members collaborated to interpret findings and socialise, have been particularly impactful. These activities allowed in-depth discussions and demonstrated that differences in knowledge are not obstacles to PPIE, but opportunities for new learning. That said, such activities are resource intensive and most involvement was conducted online, reflecting the preferred option for our regular meetings. Regardless of format, Patient and Public Partners valued being able to prepare for activities and receive feedback on how their input was utilised.

*What has helped me to stay involved is the variety of the tasks and the feedback on what then happened*,* and was produced etc. Also the flexibility to complete*,* clear indication of what was involved*,* and no pressure. [Patient Partner]*

Some also appreciated opportunities to develop their roles, leading to a noticeable shift in the balance of power, with Patient and Public Partners increasingly expressing a sense of ownership. They now confidently share decision-making responsibilities, and some have moved into leadership or training roles to support PPIE (within the BTRU and externally to support wider learning). For example, members of the team have co-facilitated training events for early career researchers, presented at conferences, attended meetings with funders and transitioned into dedicated PPIE roles within their own organisations. This has resulted in impacts that extend beyond knowledge sharing, including tangible outcomes such as co-authored articles, co-delivered events, patient-led activities, personal and professional development.

*PPIE gives us a variety of opportunities to be involved in the research as active partners*,* using our lived experiences to influence direction with a true sense of ownership in the project. It is a privilege to be able to support the people who are working so hard to improve the treatment options available to us and our input is clearly valued; we are*,* for example*,* listed as co-authors of a paper. We are helping to shape research from which so many will benefit in the future. [Patient Partner]*

Researchers’ relationships with Patients and Public Partners have also strengthened. Patient and Public Partners are not just consulted as individuals with lived experience, but are now seen as trusted partners who support mutual learning and benefit. Beyond enhancing the public-facing components of research (such as improving patient recruitment strategies or tailoring communications for lay audiences) PPIE can also help researchers leverage their knowledge. This has certainly been the case in the PRO-CAR-T™ study, where Patient and Public Partners have helped extend our collaborative networks by linking us to new patient and professional groups, amplified our messages, acted as research champions and created greater impact.

#### Areas for improvement

Patients and the public were involved at critical junctures of the PRO-CAR-T™ study and had oversight of the study through the Project Management Group. However, PPIE is time and labour intensive, and like many studies, funding constraints limited the amount of planning that could be done ahead of the bid submission stage. Moreover, while there was a commitment to co-production, many of the daily decisions, determination of priorities and scheduling arrangements were made by the academic leads, rather than patient and public contributors. Indeed, the nature of PRO research, which involves considerable data collection, complex methodologies, and protracted regulatory permissions, placed researchers under constant pressure to produce results within limited timeframes. As a result, PPIE had to be selectively prioritised, with some potential opportunities missed. For example, it may have been beneficial for Patient and Public Partners to meet the company transforming the selected PROMs into a patient app to directly inform their processes, or provide opportunities for them to take a more active role in the research (e.g., as recruiters, co-interviewers). Moreover, while the overall PPIE plan had been co-produced at the start of the project, much of timing and prioritising of activities was led by the researchers. Time constraints also meant that some activities were more consultative, rather than co-designed.

Time and resource constraints also impacted on community level engagement to increase the pool of Patient and Public members and facilitate two-way conversations. Decisions were made, primarily by the PPIE team, to focus on engagement opportunities that targeted underserved groups. These were largely identified by the PPIE team, although Patient Partners did signpost engagement opportunities that were acted upon. However, due to resources constraints and research deadlines, our PPIE efforts generally prioritised ‘involvement’ activities, over ‘engagement’. This inevitable has impacted on our ability to reach the most underserved groups, and ensure their voices are routinely heard. However, we are attempting to address these issues by working with other PPIE groups and research infrastructures to pool resources and expertise, to make it more feasible to attend engagement events and increase our reach and visibility.

## Discussion

Our experience shows that high quality PPIE is achievable in PRO research; even in the rapidly emerging area of ATMPs. Patients and the public have shown enthusiasm and willingness to be involved, offering valuable insights and skills that have significantly enhanced the research. However, evaluation of PPIE strategy for the PRO-CAR-T™ study reveals that the most challenging barriers are systematic; linked to the cultures and structures that fund and deliver PRO-research.

Our findings show that high quality PPIE requires appropriate resourcing and planning, and a collaborative approach with patients and the public. Delivering PPIE is time-consuming and requires a broad range of knowledge and skills, including: developing PPIE strategies; establishing PPIE groups and networks; building and maintaining relationships; devising varied PPIE activities; meeting support needs; monitoring requests for involvement and dissemination; training; budgeting; and evaluating impact [11, 52]. However, this work is often ‘invisible’ and overlooked in research planning [11, 52]. Projects will naturally vary in their scope and available resources, and many PRO researchers will possess strong competencies in PPIE. Nevertheless, like others, we believe that PPIE is more likely to have substantial impact when PPIE staff (e.g., academic leads for PPIE, PPIE coordinators) are seen as fundamental to the research process, adequately costed into grants [11, 52] and embedded within organisational infrastructures to promote more efficient and sustainable practices [11].

*I feel that having an organised and centralised source of guidance and direction is helping run our PPIE activities to a very high standard. I believe that this is a very important factor when PPIE is being integrated into the research activities*,* especially when they are as complex as PRO research. [BTRU staff member]*

Recognition and rewards for Patient and Public Partners is also important and recognised as best practice [54]. While most of our Patient and Public Partners were motivated by altruism, payment for time and out of pocket expenses acknowledges their status as equal partners in the co-production of knowledge (considering staff are paid for their time) and importantly, removes some financial barriers that might prevent people from being involved. However, it was also clear that other rewards are equally important, such as building friendships, acquiring new skills, sharing expertise, co-creating materials, and witnessing the impact of involvement. The latter requires timely feedback and visibly crediting patient and public contributions.

*I have valued being able to follow the project over time*,* see it develop and the interaction with the researchers. I always refer to it as “our project” [Patient Partner]*.

Like others [9, 10, 11], we advocate PPIE should happen in all PRO research, including all stages of PROM development, validation, and implementation to ensure optimal knowledge-building. However, our results provide further evidence that managing boundaries between research and PPIE can be challenging. Understandings of PPIE in PRO research varies, with language and definitions constantly evolving to reflect shifts in how roles and relationships are understood [63]. This complexity is further compounded by the similarity of methods employed in both PRO research and PPIE [13]. These challenges have also been noted in intervention development [66]. It is important, therefore, to consider if PRO activities that include patients or the public are best defined as research or PPIE, and what ethical or procedural implications they may have. While the responsibility for determining whether an activity requires ethical approval lies with those planning the activity, several tools and case-studies are available to support decision-making [13, 14, 64]. Although our experience with PRO research is UK-based, and may not reflect governance processes in other countries, understanding the ethical practices related to research and in PPIE is universally important. Indeed, others have argued that using PPIE as a substitute for qualitative research in PRO research has the potential to cross ‘ethical boundaries’ and weaken methodological rigour [63, 66]. For example, Public and Patient Partners often possess more knowledge about the research aims and PROs than typical participants and may have investments in particular outcomes. Additionally, they may not be representative of the target population, given that recruitment strategies are generally less prescriptive than those for research.

The importance of inclusive PRO research has been established [67]. However, our experience shows that reaching everyone remains challenging and reflects a widespread issue in PPIE. A survey of public contributors across NIHR-funded research showed respondents were predominantly female (57%), White British (91%), over 60 years and heterosexual [68]. Without inclusive involvement, PRO research risks reinforcing health inequities by overlooking the needs of underserved groups, including those from minority ethnic communities, and socioeconomically disadvantaged backgrounds. Frameworks such as the NIHR Race Equality Framework [69] and REP-EQUITY [70] offer practical guidance to support equitable involvement and participation in PRO research.

A significant and related challenge lies in establishing the conditions necessary for authentic co-production within institutions, such as universities. Although there is growing guidance targeted at individual researchers, structural and accepted practices within universities can inadvertently impede meaningful PPIE [71]. These barriers stem from traditionally hierarchical governance models, limited avenues for direct public accountability, inflexible ethics and reimbursement frameworks, constrained funding for sustained engagement, and an institutional emphasis on academic outputs over accessible public communication [71]. Moreover, despite widespread calls from universities, funders, and policymakers for enhanced community partnerships, there remains insufficient attention to what is required to realise these collaborations in ways that are efficient, ethical, and impactful. Although progress is being made, these persistent challenges risk fostering transactional relationships that limit genuine inclusion and mutual reciprocity.

Our work seeks to address these issues through expanded community engagement. However, given these ares are sector-wide issue, we also welcome emerging efforts that target this critical dimension of PPIE, including the NIHR Funding for Community Research Partnerships [72] and The Co-Production Futures Inquiry which is developing practical strategies to dismantle systemic barriers to co-produced research within universities and the higher education sector [73].

### Strengths and limitations

This article explores PPIE in an emerging area of PRO research. Indeed, research and the clinical use of ATMPs is expanding rapidly. It also examines PPIE from a range of stakeholder perspectives, including patients and members of the public. However, while efforts were made to adopt a systematic approach to learning, the evaluation of our PPIE was not designed as a standalone research study. Instead, it drew upon reflective practices to explore experiences and outcomes. We acknowledge that a theoretically driven study design, incorporating formal research methodologies and analytical frameworks, may have yielded additional or alternative insights. Moreover, the application of tools such as the CUBE Framework [74] could have offered deeper understanding of the relational dynamics within the study’s PPIE activities, particularly regarding the influence of power structures on engagement outcomes. The authors are also self-selected, and the manuscript may not be reflective of all individuals who have contributed to the PPIE for the PRO-CAR-T™ study. That said, our reflections do incorporate findings from a wider set of feedback, including anonymous surveys. By considering the limited literature addressing PPIE in the context of PRO research, we hope that the insights gained will contribute meaningfully to the future development and evaluation of PPIE practices in this field.

### Recommendations

Many of the challenges faced in implementing a PPIE strategy for the PRO-CAR-T™ study are deeply rooted and will take to time to resolve. Nevertheless, our experience underscores the importance of researchers striving for excellence in PPIE practice. Table 3 presents a comprehensive set of practical recommendations for effectively undertaking PPIE in PRO research, based on insights from conceptualising and operationalising a strategy for the PRO-CAR-T™ study. Given the complexity and evolving nature of PRO studies, particularly those involving ATMPs, these recommendations emphasise the importance of early planning, inclusivity, meaningful collaboration, and ongoing evaluation. The recommendations encompass preparatory steps such as familiarisation with best practice resources and securing appropriate support, strategies to foster diverse and accessible inclusion, and approaches to ensure that contributions are valued and have tangible impact. By integrating these principles throughout the research cycle, teams can enhance both the quality of PRO research and the experiences of patient and public contributors.

**Table 3:** Practical recommendations for undertaking PPIE in PRO research

**Make it possible**: The groundwork for PPIE is best done before research starts. This is particularly useful in PRO research focusing on ATMPs, as there is limited evidence about the experiences of patients and patient populations can be small.
• **Understand best practice** in PPIE using introductory guides such as the NIHR Briefing Notes for researchers and those specific to ATMP research, such as ATMP Engage (provided by the European Consortium for Communicating Gene and Cell Therapy Information - EuroGCT). • **Seek support** from local PPIE experts and funding infrastructures, such as the NIHR Research Support Service Hub. • **Look for PPIE training opportunities.** Many research organisations and infrastructures for PPIE offer or signpost in-person, online and recorded training opportunities. • **Integrate PPIE fully** by understanding how PPIE is relevant across the research cycle: from developing the research questions, applying for funding and ethical approval, sitting on advisory groups, carrying out the research and disseminating the research findings. • **Identify your target patient and public groups**, using frameworks to consider who might be involved, and develop links with local PPIE, patient, carer, community or clinical groups to build mutually beneficial relationships. • **Familiarise yourself with PPIE funding landscape**, as some funders, research infrastructures and employing organisations offer small ‘starter’ grants to support pre-funding PPIE and post-research ‘impact’ funding. • **Work with patients and the public to develop grant submissions**,** research proposals and PPIE plans**, using generic planning tools, online planners, generic frameworks to incorporate PPIE into PRO measure development and specific tools for PPIE in the development of clinical trial protocols, as applicable. • **Ensure adequate costs** for PPIE staff, PPIE activities, remuneration of Patient and Public contributors, community partnerships. Use best practice guidelines such as the NIHR Payment guidance and cost calculator. This should include costs for PPIE staff, where possible and reflect important competencies.
**Make it inclusive**: PPIE is better when diverse voices are included and heard. This means building relationships with varied communities and offering appropriate support.
• **Make a commitment to Equality**,** Diversity**,** Inclusion (EDI)**, using resources such as NIHR Race Equality Framework to improve involvement of diverse communities and use practical guidance to being inclusive and building relationships within communities. *[N.B., PRO-research involving patients are also recommended to use REP-EQUITY toolkit to guide recruitment]* • **Co-design PPIE activities** with patients and the public to ensure they are likely to be acceptable, feasible and deliver meaningful outcomes. • **Discuss personal requirements** with Patients and Public Partners at the outset and ensure accessibility is considered when planning activities (e.g., accessibility information provided for venues, use plain language and accessibility checkers to ensure documents and presentations are accessible to people with disabilities). • **Offer choice and flexibility where possible.** Plan varied involvement activities to suit different preferences, using a range of formats, venues and times (e.g., online meetings, in-person meetings, interactive workshops, at-home tasks, digital noticeboards, simple polls). Where possible, offer multiple ways to be involved in an activity (e.g., hybrid meetings, information exchanged by email, hard copy, verbally to suit preferences).
**Make it rewarding**: PPIE works better when people feel valued and can see the impact of their contributions.
• **Adopt a co-production model of PPIE.** Don’t just consult people, but make commitment to share power and decision-making throughout the project. There are many guidance documents and practical examples of how patients and the public can work as partners at all levels of research and projects. • **Help patients and the public understand PPIE and their roles**, using helpful materials such as the NIHR Starting Out Guide and provide user-friendly terms of reference. • **Have a payment policy for PPIE and offer timely payments** for time and reasonable out of pocket expenses, with support to gain advice if these are likely to impact on their tax or welfare benefit. Guidance is often available for both researchers and public contributors, including easy read guidance. • **Give adequate time and information to be involved with PPIE activities.** Create advertisements/invitations that enable informed decision-making (ideally sent 1 month before activities) and pre-activity packs to facilitate understanding and involvement (ideally sent 7–10 days beforehand). It can be helpful if presentations or materials are sent in advance of meetings. • **Provide timely feedback and updates.** These should offer thanks and an explanation of how contributions are used (or reasons why they may not be acted upon). Practical tips to structure and share feedback are available. • **Regularly review PPIE.** Invite anonymous feedback after PPIE activities and make ‘working together’ a regular feature of PPIE and management meetings – allowing different opportunities to reflect on experiences and impact and suggest improvements. • **Value contributions.** In addition to payment, ensure contributions are formally acknowledged in ways that suit Patient and Public Partners, including authorship, named acknowledgments, using photos (with permission) to showcase personal involvement. Encourage and support patient-led activities and personal development (e.g., new skills).
**Make it count**: PPIE should add value to the PRO research being undertaken and support personal development.
• **Ensure patients and the public are part of your governance**,** management and steering** structures, to ensure all decisions are patient-centred and to support transparency and accountability. • **Choose meaningful outcomes.** Work together with Patient and Public Partners to choose meaningful outcomes of PPIE and how to disseminate them. • **Keep an impact log** to record what you have done, and what impact this is making, such as the PIRIT (Public Involvement in Research Impact Toolkit) to help track patients and public contributions and the difference they make to the research. • **Monitor EDI** to identify and address under-representation. • **Report PPIE appropriately**. For example, include PPIE in all reports and publications. Tools such as the GRIPP 2 provide international guidance for reporting PPIE in health and social care research. • **Share your learning**: Promote efficiency, shared learning and equity in PPIE by sharing your knowledge and contributing to debates in PPIE and initiatives to promote a more harmonised approach across PRO research. Resources can be shared on centralised resources like the NIHR Learning for Involvement website.

These can be read alongside other recommendations for incorporating PPIE into the development of PROMs [9], their use in clinical trials [12] and the wider organisational infrastructure for PRO research [11].

## Conclusions

The complexity and novelty of ATMPs, such as CAR-T, present distinct challenges for PPIE. The pool of individuals with lived experience remains relatively small, and many are undergoing intensive treatment or managing long-term effects, which can make sustained involvement difficult. Discussions are often emotionally charged, and the gravity of these conditions means that the death of PPIE contributors is a real possibility; something our team has sadly experienced firsthand. Yet, these same complexities also create powerful motivations and opportunities. Individuals with direct experience of CAR-T often demonstrate deep motivation to contribute, offering invaluable insights into outcomes that matter most to patients and factors that complicate the collection of PRO data, such as cytokine release syndrome, neurotoxicity, and fatigue. Importantly, we found that contributors with broader but related experiences, such as those affected by conventional therapies or donors, can also enrich the research. Their perspectives were often driven by a desire to accelerate the development of more effective and compassionate treatments, promote equitable access, and improve public understanding of these therapies. Integrating this diversity into PRO research ensures that patient experiences more fully inform therapy development, trial design, and health system preparedness, while also addressing equity concerns. As CAR-T therapies expand beyond hematologic malignancies into solid tumours and other conditions, there is a clear opportunity to transfer this learning. As such, we firmly believe that PPIE (alongside the different and distinct activity of research participation) is an essential component that should be included in future funding strategies for ATMPs and PRO research.

## Data Availability

Not applicable. Manuscript does not contain any data.
